# A Case of Fatal Rhino-Orbital Mucormycosis Associated With New Onset Diabetic Ketoacidosis and COVID-19

**DOI:** 10.7759/cureus.13163

**Published:** 2021-02-05

**Authors:** Salomon Waizel-Haiat, Jose Alberto Guerrero-Paz, Luis Sanchez-Hurtado, Salvador Calleja-Alarcon, Laura Romero-Gutierrez

**Affiliations:** 1 Otolaryngology, Hospital de Especialidades “Dr. Bernardo Sepúlveda Gutiérrez” Centro Médico Nacional Siglo XXI, Mexico City, MEX; 2 Critical Care Medicine, Hospital de Especialidades “Dr. Bernardo Sepúlveda Gutiérrez” Centro Médico Nacional Siglo XXI, Mexico City, MEX

**Keywords:** rhinosinusitis, covid-19, mucormycosis, diabetic ketoacidosis (dka), fungal rhino sinusitis

## Abstract

Mucormycosis is an invasive fungal infection, often acute and extremely severe, occurring in patients with an underlying condition. Coinfection in patients with coronavirus disease 2019 (COVID-19) has been reported, often bacterial. A 24-year-old female is presented with acute fatal rhino-orbital mucormycosis and COVID-19.

We report one of the first cases of rhino-orbital mucormycosis and COVID-19. With this case, we highlight the importance of considering mycotic coinfection in COVID-19 patients with diabetes.

## Introduction

Mucormycosis (Zygomycosis) is an invasive fungal infection, often acute and extremely severe caused by opportunist and ubiquitous fungi belonging to the class Phygomycetes, subclass Zygomycetes, order Mucorales, family Mucoraceae; usually by the following species: *Absidia corymbifera*, *Apophysomyces elegans*, *Cunninghamella bertholletiae*, *Mucor rouxii*, *Rhizomucor pussillus*, *Rhizopus arrhizus*, and by species of the genus Saksenaea spp. The species mentioned above suppose the third cause of invasive fungal infection after Aspergillus and Candida spp in humans [1].

It is acquired by the establishment or implantation of the fungal spores in the oral, nasal and conjunctival mucosa (rhino-orbito-cerebral), by inhalation (pulmonary), or by the ingestion of contaminated food (digestive); as they quickly colonize nutriments rich in simple carbohydrates being glucose its main energy source [2].

Coinfection in patients with coronavirus disease 2019 (COVID-19) has been reported on multiple series, being bacterial in origin the most frequent; and fungal infection being reported only in severe cases [3-5].

Here we present a case of rhino-orbital mucormycosis associated with ketoacidosis secondary to recent-onset diabetes mellitus and infection with severe acquired respiratory syndrome coronavirus 2 (SARS COV-2).

## Case presentation

A 24-year-old female, resident of Mexico City, with past medical history of obesity and being exposed to family members with COVID-19, presented to the ED of our tertiary care center with respiratory failure and oxygen saturation of 80%, given the findings she was managed in accordance with national and international guidelines.

Her family reported she began with pain in the left midface region six days prior, two days later she developed progressive left lid swelling and maxillary hypoesthesia; therefore, a primary care physician suspected infection, beginning treatment with oral antibiotics (amoxicillin-clavulanate 875/125 mg twice-daily) with partial remission of pain. However, on subsequent days she continued with progression of soft-tissue edema, being referred to our center for an extended study protocol and management.

Physical examination revealed severe left lid edema with extension to the upper lip and malar region, left proptosis with a hyperemic conjunctiva, and an opaque cornea which precluded further evaluation. Rhinoscopy revealed edema of the left nasal mucosa with an impaired response to vasoconstriction, without evidence of necrotic tissue or purulent discharge. Samples for a direct exam were taken. The oral cavity examination only revealed pallor of hard palate mucosa. We could not assess sensitivity of facial structures due to the patient being sedated.

**Figure 1:**
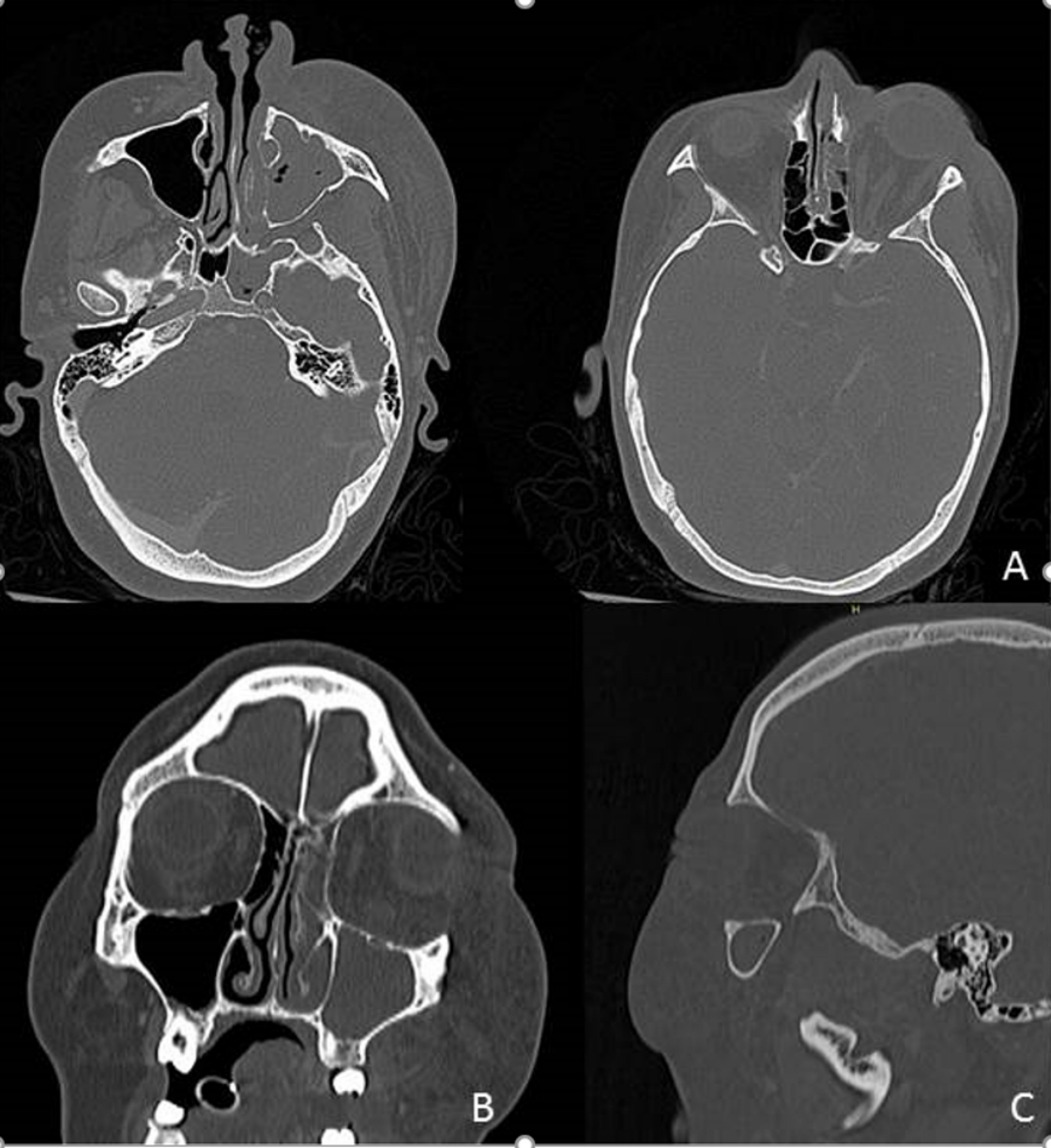
Contrast enhanced CT showing occupancy of the left maxillary, ethmoid, and sphenoid sinuses, without apparent intracranial involvement. Striation of the extraconal fat is observed in the lower left region, as well as periorbital and ipsilateral facial region with associated proptosis. Axial (A), coronal (B) and sagittal (C).

A contrast-enhanced CT of head and chest was performed revealing soft tissue swelling of the left inferior turbinate and thickening of the mucosa of the maxillary, ethmoid, and sphenoid sinuses on the ipsilateral side. Periorbital and midfacial structures of the left sides also had soft tissue swelling, with associated proptosis (Figure 1).

**Figure 2:**
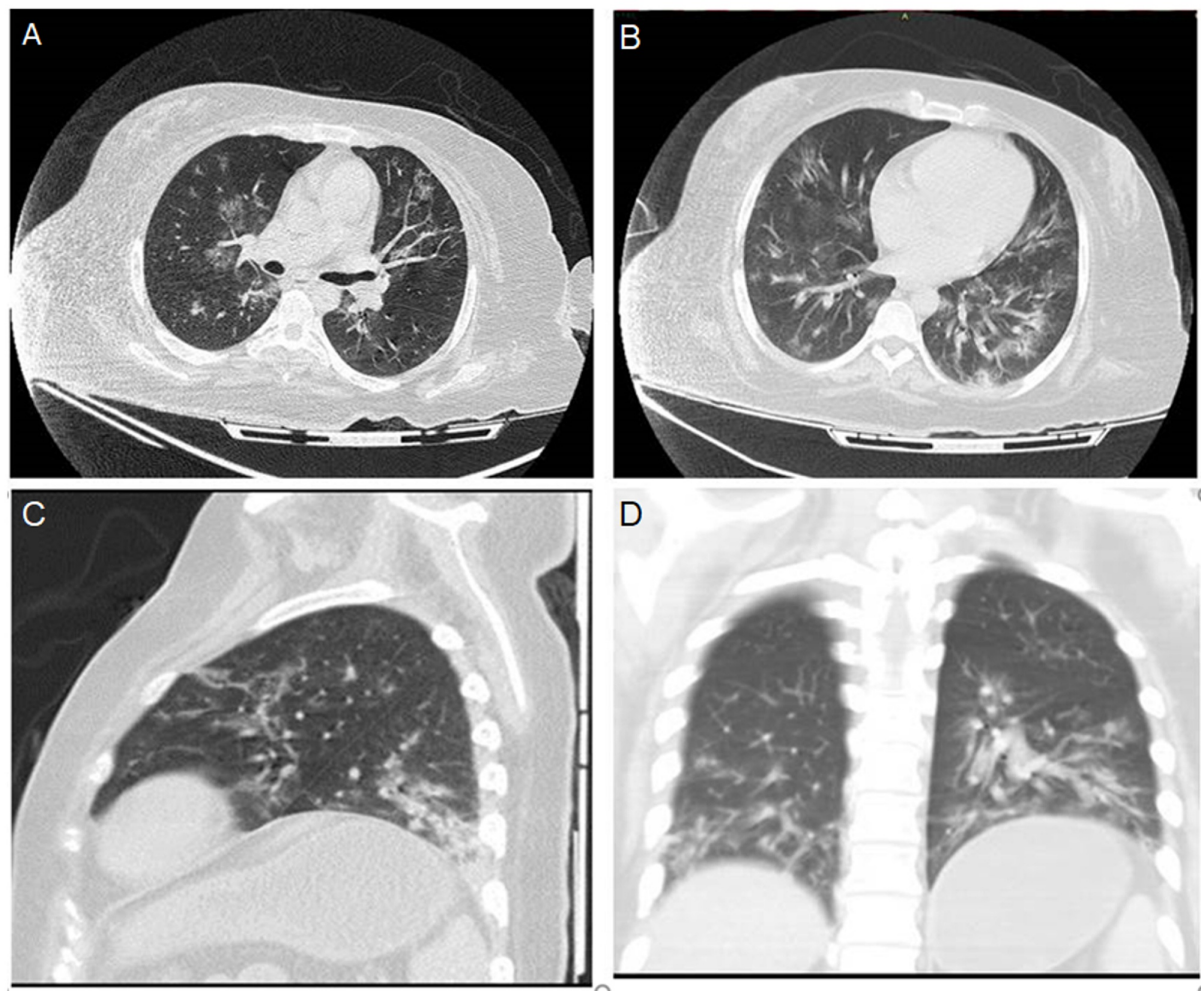
CT of the chest with multiple areas of increased density in a ground-glass pattern with diffuse bilateral distribution with peripheral predominance coexisting with consolidation in lower lobes and intralobular septal thickening. Axial mainstem bronchii level (A), Axial right pulmonary artery level (B), Sagittal (C), Coronal (D)

The thorax section had findings suggestive of atypical pneumonia due to SARS COV-2, consisting of multiple parenchymatous zones with an increased density and bilateral distribution with peripheral predominance giving a ground-glass appearance, coexisting with consolidation of lower lobes (Figure 2). 

We present the ancillary studies performed during her management at our center (Table 1).

**Table 1:** Ancillary tests during patient hospitalization. SARS COV-2 RT-PCR: Severe acquired respiratory syndrome coronavirus 2 reverse-transcriptase polymerase chain reaction

Parameter	Day 1	Day 2	Day 3
Glucose	509 mg/dL	455 mg/dL	331 mg/dL
Urea	25.7 mg/dL	42.8 mg/dL	107.0 mg/dL
Creatinine	1.07 mg/dL	0.92 mg/dL	2.95 mg/dL
Alanine transaminase	10 u/L	14 u/L	108 u/L
Aspartate transaminase	26 u/L	34 u/L	31 u/L
Lactic dehydrogenase	373 u/L	289 u/L	1395 u/L
Albumin	4.9 g/dL	2.9 g/dL	-
Potassium	4.50 mEq/L	3.3 mEq/L	7.80 mEq/L
Sodium	127 mEq/L	145 mEq/L	151 mEq/L
Leucocytes	23 x 10^3/µL	23 x 10^3/µL	16 x 10^3/µL
Neutrophils	20.58 x 10^3/µL	21.68 x 10^3/µL	13.2 x 10^3/µL
Platelets	398 x10^3/µL	302 x10^3/µL	64 x 10^3/µL
Prothrombin time	18.4 s	17.7 s	26.4 s
Fibrinogen	1113.00 mg/dL	-	1029 mg/dL
D- Dimer	3.12 µg/mL	-	15.88 µg/mL
Urinalysis	pH 5, density 1.025, proteins 150 mg/dL, glucose 1000 mg/dL, Ketones 150 mg/dL	-	-
Arterial gasometry	pH 6.9, pCO2 23.3, HCO3 4.7, Anion gap 20.3	-	-
Procalcitonin	-	23.66 ng/mL	-
Central hemoculture	No growth		
SARS COV-2 RT-PCR	-	Positive	-
Tracheal secretion culture			Klebsiella pneumoniae, Enterobacter cloacae, Enterococcus faecalis

Our initial diagnosis was complicated rhinosinusitis of probable fungal origin, with the following associated diagnoses: severe diabetic ketoacidosis, diabetes mellitus, severe metabolic acidosis, and highly likely atypical pneumonia due to SARS COV-2. Due to severity of infection, we started imipenem/linezolid and amphotericin B as empirical treatment.

**Figure 3:**
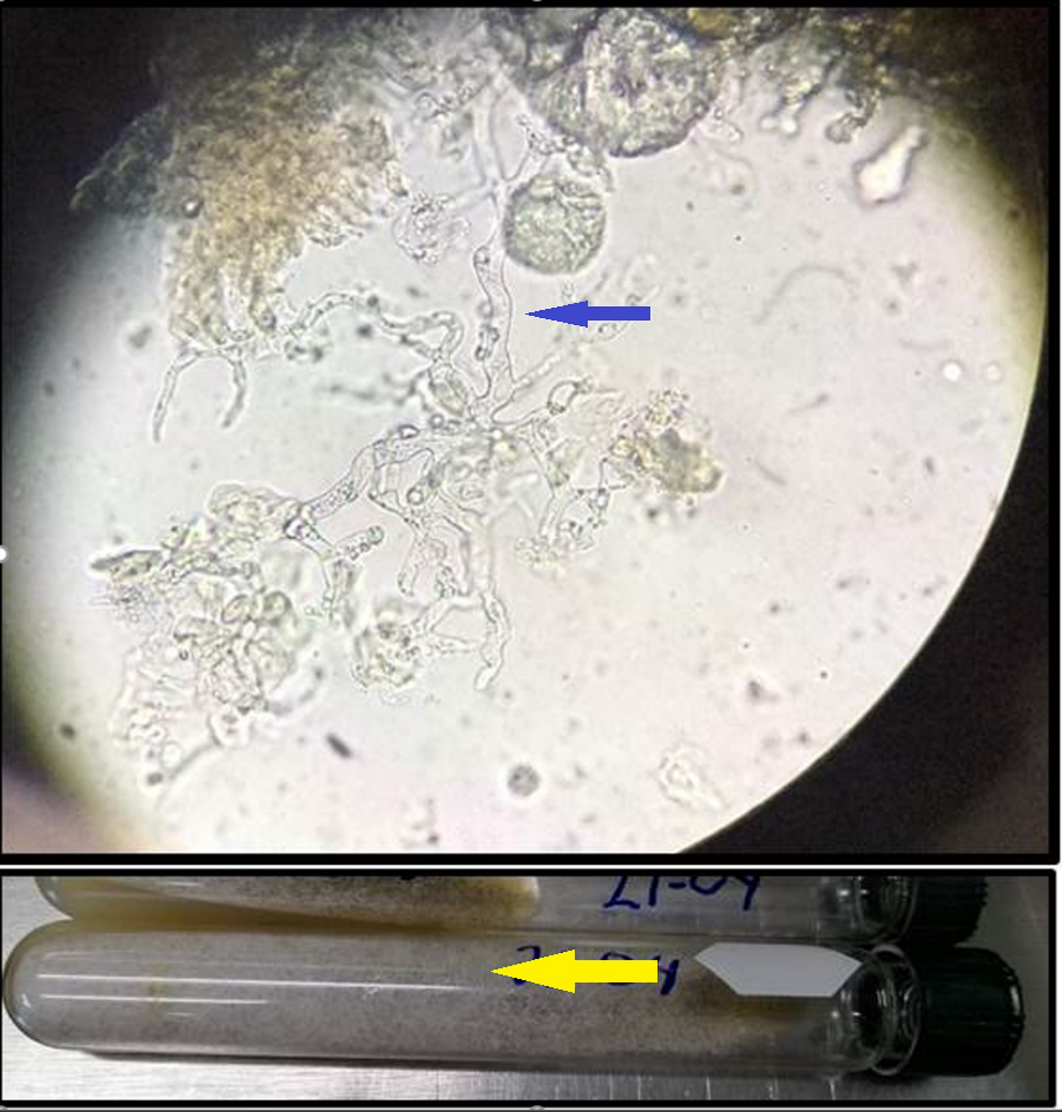
Upper image showcasing direct exam with nonseptate, thick hyphae (blue arrow), lower image showcasing philamentous floccose white colonies (yellow arrow).

Direct exam was repeated due to suspicion of a false negative, confirming diagnosis in the next sample, with further culture in Sabouraud media isolating Lichteimia (Absidia) spp (Figure 3). COVID-19 was confirmed with reverse transcriptase-polymerase chain reaction (RT-PCR) of bronchioalveolar fluid (Logix Smart™, Co-Diagnostics, Inc., Salt Lake City, UT, USA).

**Figure 4:**
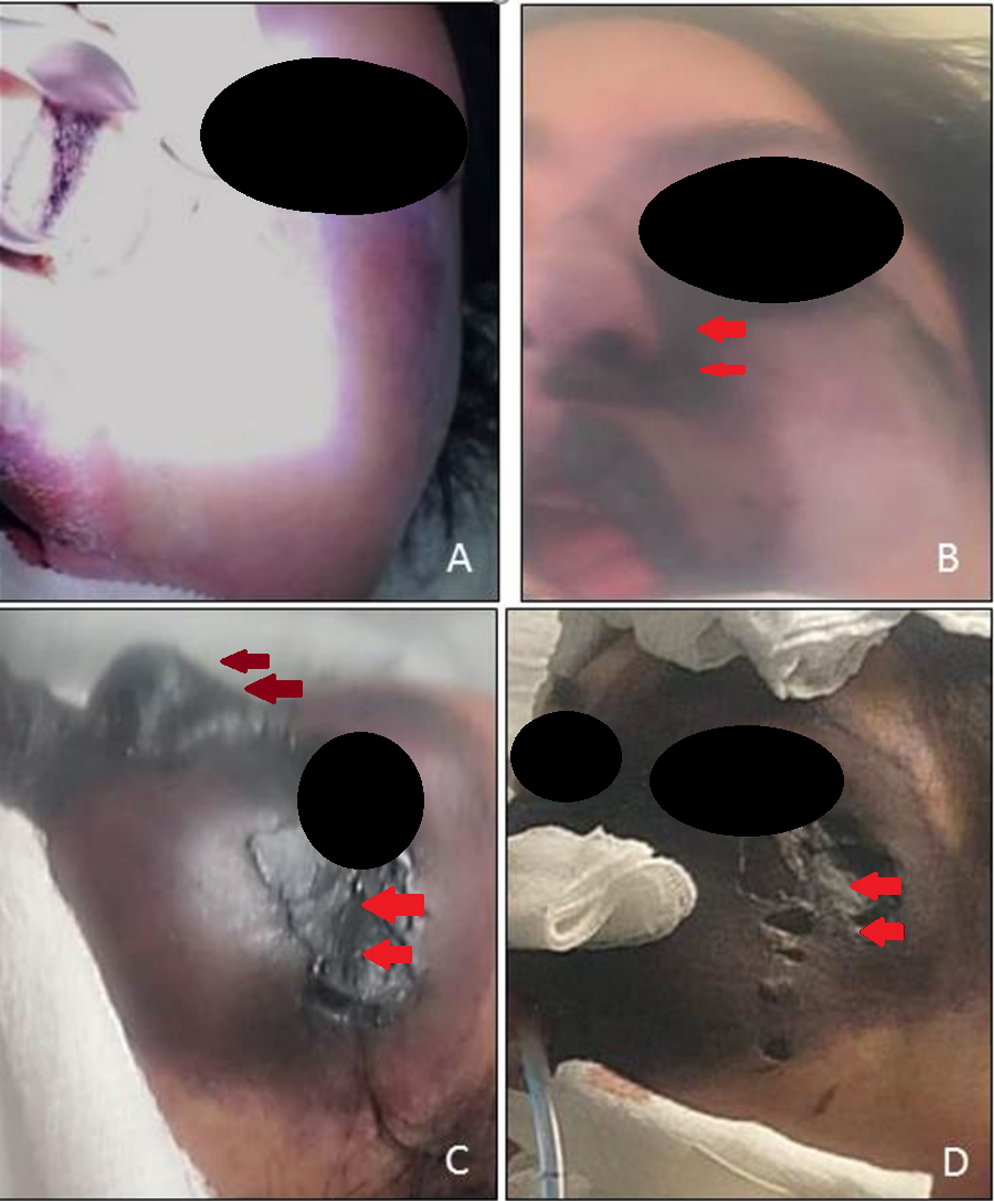
Clinical pictures demonstrating the rapid fatal evolution. (A) Admission, (B) first day, (C) second day, (D) third day. Lower lid erythema with progression to a eschar with progressive expansion (red arrows), with further extension to the nasal soft tissues (dark red arrows). Permission was granted by the patient family to reproduce the clinical photographs.

Even with the established management of the severe diabetic ketoacidosis and mechanical ventilation, the patient had an unfavorable evolution due to refractory metabolic acidosis combined with the pulmonary insult and aggregated acute kidney injury due to disseminated intravascular coagulopathy, which precluded further surgical management, dying of multi-organic failure due to unresponsive septic shock (Figure 4).

## Discussion

Mucormycosis is an angioinvasive infection. It is categorized in rhinocerebral, cutaneous, disseminated, gastrointestinal, or pulmonary [6]. In a meta-analysis of 600 series with 851 cases, diabetes mellitus is being reported as the most common underlying condition and an independent factor of rhino-orbital-cerebral mucormycosis, being Rhizopus spp the most common in this group, with an overall mortality of 46% [7]. Despite these findings, there has been a global increase in cases associated with hematologic neoplasms and organ transplant recipients [8].

In Mexico, diabetes mellitus is also the most common underlying condition with 72% of the cases reported, of which 75% had rhinosinusitis, with an overall mortality of 51% [9].

Baker, an American pathologist retook and coined the term Mucormycosis in 1957 for aggressive infection by Rhizopus [10].

The pathogenic mechanisms implicated in the fungal aggressiveness are the decrease of phagocytic function, the more available iron due to displacement of protons by transferrin in diabetic ketoacidosis and the fungal heme oxygenase which facilitates iron uptake for its metabolism [11].

Diagnosis requires rapidness and high index of suspicion due to fulminant progression; the following red flags have been proposed in diabetic patients: cranial nerve palsy, diplopia, mid-facial pain, proptosis, periorbital edema, apex orbital syndrome, and palatine ulcer [6].

According to international guidelines, management must be in a tertiary care center to provide multidisciplinary approach. It is recommended to perform imaging and complimentary endoscopy. If orbit or brain extension, MRI is acquired. Finally, CT imaging should be repeated in unstable patients. Direct exam and histopathology confirm diagnosis. Treatment consists of aggressive surgical debridement, repeating as necessary with adjuvant liposomal amphotericin B (10 mg/kg) [8].

In actual literature there has been reported three rhino-orbitary cases associated with COVID-19 [12-14]. We consider it important to identify the association given the complications and modifications of thrombotic, pulmonary, and metabolic markers both entities share. In our opinion, the immune mechanisms seem to be different due to the fact mycotic dissemination is dependent mostly on modification of phagocytic function; further studies should be performed to determine if this group of patients have further immune dysregulation.

## Conclusions

The COVID-19 pandemic precludes access to pathologies not related with this entity. Due to the high index of suspicion required, late diagnosis in association with an acute respiratory distress syndrome predisposes to a catastrophic scenario. We consider important to report this case to warn of the possibility of COVID-19 as a trigger of diabetic ketoacidosis which could predispose an invasive fungal infection or another high-risk disease. Lastly, we emphasize the hardship of therapeutic decisions in this group of patients with severe coinfections.

In our opinion, the severe immunosuppressive state secondary to diabetic ketoacidosis without previous treatment made the patient susceptible to both severe COVID-19 and mucormycosis.

## References

[1] Martínez-López R (2005). Ecología de los hongos patógenos para el hombre. Rev Mex Mic.

[2] Martínez-López R (1984). Estudio de hongos atmosféricos en un medio hospitalario. Gac Med Mex.

[3] Lansbury L, Lim B, Baskaran V, Lim WS (2020). Co-infections in people with COVID-19: a systematic review and meta-analysis. SSRN Electron J.

[4] Zhu X, Ge Y, Wu T (2020). Co-infection with respiratory pathogens among COVID-2019 cases. Virus Res.

[5] Song G, Liang G, Liu W (2020). Fungal co-infections associated with global COVID-19 pandemic: a clinical and diagnostic perspective from China. Mycopathologia.

[6] Suganya R, Malathi N, Karthikeyan V, Janagaraj VD (2019). Mucormycosis: a brief review. J Pure Appl Microbiol.

[7] Jeong W, Keighley C, Wolfe R (2019). The epidemiology and clinical manifestations of mucormycosis: a systematic review and meta-analysis of case reports. Clin Microbiol Infect.

[8] Cornely OA, Alastruey-Izquierdo A, Arenz D, Chen SCA, Dannaoui E, Hochhegger B; Mucormycosis ECMM MSG Global Guideline Writing Group (2019). Global guideline for the diagnosis and management of mucormycosis: an initiative of the European Confederation of Medical Mycology in cooperation with the Mycoses Study Group Education and Research Consortium. Lancet Infect Dis.

[9] Corzo-León DE, Chora-Hernández LD, Rodríguez-Zulueta AP, Walsh TJ (2018). Diabetes mellitus as the major risk factor for mucormycosis in Mexico: epidemiology, diagnosis, and outcomes of reported cases. Med Mycol.

[10] Baker RD (1957). Mucormycosis-a new disease?. J Am Med Assoc.

[11] Ibrahim AS, Spellberg B, Walsh TJ, Kontoyiannis DP (2012). Pathogenesis of mucormycosis. Clin Infect Dis.

[12] Mekonnen ZK, Ashraf DC, Jankowski T (2020). Acute invasive rhino-orbital mucormycosis in a patient with COVID-19-associated acute respiratory distress syndrome. Ophthalmic Plast Reconstr Surg.

[13] Mehta S, Pandey A (2020). Rhino-orbital mucormycosis associated with COVID 19. Cureus.

[14] Werthman-Ehrenreich A (2020). Mucormycosis with orbital compartment syndrome in a patient with COVID-19. Am J Emerg Med.

